# Sjögren Syndrome Complicated by Mucosa-Associated Lymphoid Tissue Lymphoma and Lymphocytic Interstitial Pneumonia

**DOI:** 10.3389/fonc.2015.00179

**Published:** 2015-08-06

**Authors:** Fatma Ahmed, Osama Raslan, Razi Muzaffar, Nadeem Parkar, Nitin Marwaha, Medhat M. Osman

**Affiliations:** ^1^Saint Louis Univeristy, Saint Louis, MO, USA

**Keywords:** Sjögren syndrome, MALT lymphoma, LIP, FDG PET/CT, FDG avid

## Abstract

Sjögren syndrome (SS) is an autoimmune disease with exocrine glands dysfunction and multiorgan involvement. It is associated with increased risk of lymphoproliferative disorders, especially B-cell marginal zone lymphoma. While the role of F-18 Fluorodeoxyglucose position emission tomography/computed tomography (F-18 FDG PET/CT) for evaluation of lymphoma has been established, its use in patients with a chronic history of SS to evaluate for possible lymphoproliferative disorders or multiorgan involvement is limited. We present a case of chronic SS in which F-18 FDG PET/CT demonstrated FDG avid intraparotid and cervical lymph nodes pathologically proven to be mucosa-associated lymphoid tissue lymphoma. In addition, the patient had bibasilar cystic changes consistent with lymphocytic interstitial pneumonia.

## Introduction

F-18 Fluorodeoxyglucose position emission tomography/computed tomography (F-18 FDG PET/CT) plays an important role in the evaluation of lymphoma. Patients with chronic Sjögren syndrome (SS) are at risk of lymphoproliferative disorders, particularly B-cell marginal zone lymphoma. There are limited publications on the role of F-18 FDG PET/CT in evaluating these patients for possible development of lymphoma or multiorgan involvement with only a few cases reported. We present a patient with a 15-year history of SS in which F-18 FDG PET/CT demonstrated hypermetabolic intraparotid and cervical lymph nodes pathologically proven to be lymphoma. In addition, there were bibasilar cystic changes in the lungs consistent with lymphocytic interstitial pneumonia (LIP).

## Background

**Figure 1:**
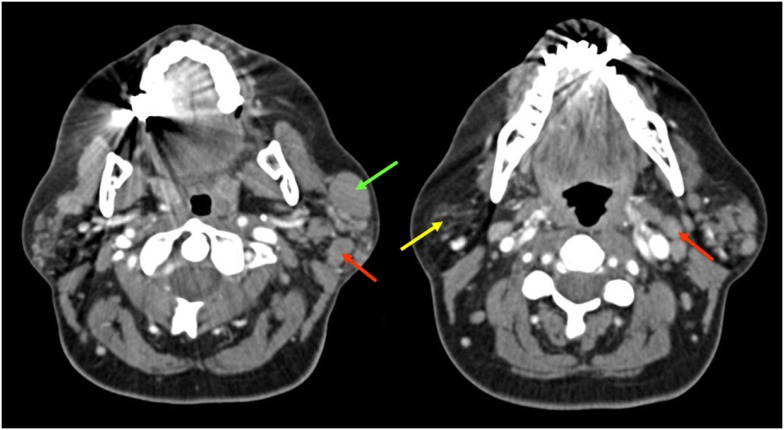
**Contrast-enhanced computed tomography demonstrated fatty atrophy of the parotid glands (yellow arrow), enhancing predominately left parotid nodule (green arrow), and enlarged cervical lymph nodes (red arrows)**.

**Figure 2:**
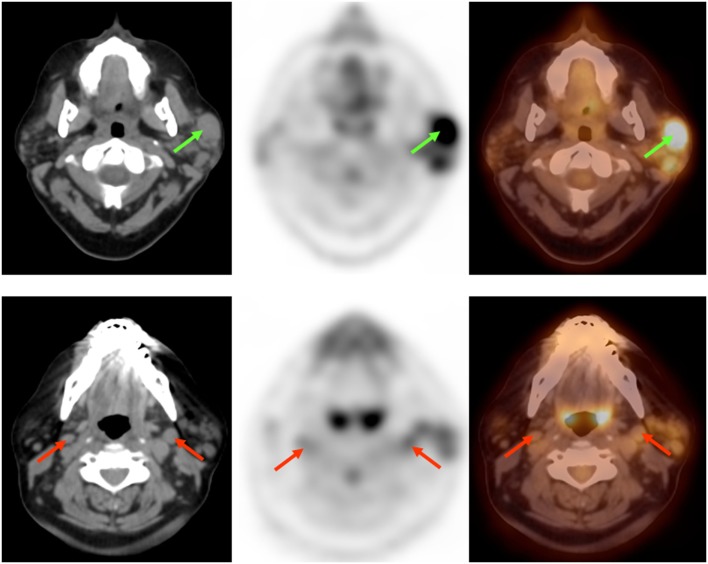
**F-18 Fluorodeoxyglucose position emission tomography/computed tomography demonstrated FDG avid predominately left parotid nodule (green arrows) and cervical lymph nodes (red arrows), suspicious for lymphoma**.

**Figure 3:**
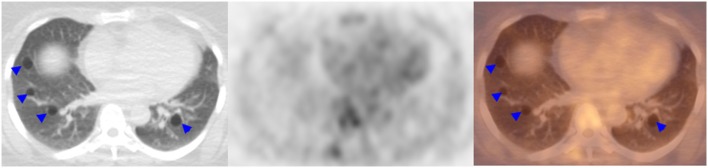
**F-18 Fluorodeoxyglucose position emission tomography/computed tomography demonstrated bibasilar thin-walled lung cysts, consistent with LIP (blue arrowheads)**.

**Figure 4:**
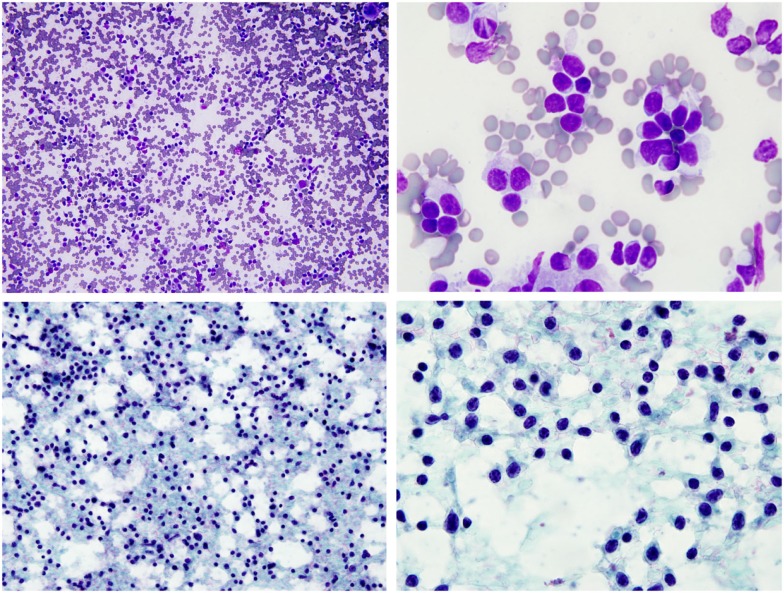
**FNA of left parotid nodule demonstrated monotonous population of small to intermediated size atypical lymphocytes**. (Top panels – Diff-Quik stain, bottom panels – Pap stain).

A 51-year-old female with a history of SS diagnosed 15 years ago presented with a mass on her left face. Contrast-enhanced computed tomography (CCT) demonstrated fatty atrophy of the parotid glands (Figure 1, yellow arrow), predominately enhancing left parotid nodule (Figure 1, green arrow), and enlarged left cervical lymph nodes (Figure 1, red arrows). Diagnostic considerations included chronic sialadenitis but lymphoma or lymphoproliferative disorder was not excluded. F-18 FDG PET/CT demonstrated an FDG avid left parotid nodule with SUV Max 8.6 (Figure 2, green arrows) and cervical lymph nodes (Figure 2, red arrows), which were suspicious for lymphoma. Other considerations of FDG lesions in the salivary glands may include salivary gland tumors or lymph node metastases from squamous cell carcinoma although these would be less likely in this particular patient. In addition, CT lung window of the F-18 FDG PET/CT showed non-FDG avid bibasilar thin-walled lung cysts, consistent with LIP (Figure 3, blue arrowheads). CT guided fine needle aspiration of the left parotid gland nodule demonstrated medium to intermediate size atypical lymphocytes. The atypical lymphocytes were found to be positive for PAX5 and negative for CD3, CD10, and CD5 with kappa restriction on flow cytometric analysis. On histological evaluation, a lymphoepithelial lesion with atypical lymphocytes of above mentioned immunophenotype is noted and a diagnosis of extranodal marginal zone lymphoma of mucosa-associated lymphoid tissue (MALT lymphoma) was rendered (Figure 4). This case demonstrates SS-associated LIP complicated by MALT lymphoma. The patient would be followed monthly and treatment options included single agent Rituxan or an immunochemotherapy regimen, such as bendamustine/rituximab. The patient is currently receiving hydroxychloroquine therapy for SS and monitoring of lymphoma, also follow-up F-18 FDG PET/CT was recommended per the oncologist.

## Discussion

Sjögren syndrome is an autoimmune condition characterized by lymphocytic infiltration of exocrine glands and multiorgan involvement that affects approximately 0.1–3.0% of the general population (female/male ratio 9:1) (1). SS is complicated by lymphoproliferative disorders, most commonly B-cell lymphoma, in 5–10% of patients with two to eight times an increased risk of death compared to the control population (2).

In patients with SS thoracic involvement, airway disease and interstitial lung disease (ILD) are common. The most observed histological patterns are non-specific interstitial pneumonia (NSIP) and LIP, while usual interstitial pneumonia (UIP) is less frequent (3). Ryoko et al. described CT findings of diverse thoracic manifestation of SS with its pathologic correlation. They divided the pulmonary manifestation into three groups: interstitial pneumonia, airway abnormalities, and lymphoproliferative disorders (4).

In a recent study, Cohen et al. developed a PET/CT activity score which integrated PET for the work-up of SS patients and associated lymphoma to correlate with disease activity. They found that patients with SS had a significantly higher FDG uptake in salivary glands, lymph nodes, and lungs compared to control group. Furthermore, patients who developed lymphoma had an even higher SUV max than patients without lymphoma. They concluded F-18 FDG PET/CT could be useful for optimizing diagnosis, post-therapy evaluation of lymphoma, and multiorgan involvement in patients with SS. It is especially helpful if combined with anatomic imaging and minimally invasive techniques for histological diagnosis (5).

## Conclusion

F-18 Fluorodeoxyglucose position emission tomography/computed tomography can be useful for optimizing diagnosis, post-therapy evaluation of lymphoma, and multiorgan involvement in patients with SS. It is particularly helpful when combined with anatomic imaging and minimally invasive techniques for histological diagnosis (5).

## Conflict of Interest Statement

The authors declare that the research was conducted in the absence of any commercial or financial relationships that could be construed as a potential conflict of interest.
